# Development of an Effective Sonotrode Based Extraction Technique for the Recovery of Phenolic Compounds with Antioxidant Activities in Cherimoya Leaves

**DOI:** 10.3390/plants11152034

**Published:** 2022-08-04

**Authors:** Beatriz Martín-García, María José Aznar-Ramos, Vito Verardo, Ana María Gómez-Caravaca

**Affiliations:** 1 Department of Analytical Chemistry, Faculty of Sciences, University of Granada, Avd. Fuentenueva s/n, 18071 Granada, Spain; 2 Department of Nutrition and Food Science, University of Granada, Campus of Cartuja, 18071 Granada, Spain; 3 Biomedical Research Center, Institute of Nutrition and Food Technology ‘José Mataix’, University of Granada, Avda del Conocimiento sn., Armilla, 18100 Granada, Spain

**Keywords:** cherimoya leaves, Box-Behnken design, phenolic compounds, proanthocyanidins, HPLC-MS

## Abstract

The leaves of *Annona cherimola* Mill (cherimoya) are a potential source of phenolic compounds that have been shown to have beneficial properties. Therefore, this study focuses on establishing an ultrasonic-assisted extraction of phenolic compounds in cherimoya leaves using a sonotrode. For that purpose, a Box-Behnken design based on a response surface methodology (RSM) was used to optimize factors, such as amplitude, extraction time and solvent composition to obtain the maximum content of phenolic compounds by HPLC-MS and the maximum in-vitro antioxidant activity by DPPH, ABTS and FRAP assays in ‘Fino de Jete’ cherimoya leaves. The optimal conditions were 70% amplitude, 10 min and 40:60 ethanol/water (EtOH/H_2_O) (*v*/*v*). The results obtained under these optimum conditions by using a sonotrode were compared with those from an ultrasonic bath; briefly, recovery of phenolic compounds by sonotrode was 2.3 times higher than a bath. Therefore, these optimal conditions were applied to different varieties ‘Campas’, ‘Fino de Jete’ and ‘Negrito Joven’ harvested in the Tropical Coast of Granada (Spain). A total of 39 phenolic compounds were determined in these cherimoya leaf extracts, 24 phenolic compounds by HPLC-MS and 15 proanthocianidins by HPLC-FLD. 5-p-coumaroylquinic acid, lathyroside-7-O-α-l-rhamnopyranoside and quercetin hexose acetate were first identified in cherimoya leaves. The most concentrated phenolic compounds were the flavonoids, such as rutin and quercetin hexoside and proanthocyanidins including monomers. Almost no significant differences in the phenolic content in these cultivars were found (11–13 mg/g d.w. for phenolic compounds and 11–20 mg/g d.w. for proanthocyanidins). In addition, sonotrode ultrasonic-assisted extraction has been shown to be an efficient extraction technique in the phenolic recovery from cherimoya leaves that could be implemented on an industrial scale.

## 1. Introduction

The Annonaceae family has 112 genera and comprises around 2500 species. Most of these species are found in the tropics [1]. This family comprises four subfamilies: Anaxagoreoideae, Ambavioideae, Annonoideae and Malmeoideae and 14 tribes [2]. A few of these species belong to just two genera *Annona L.* and *Asimina Adans.* provides edible fruits, which are mainly found in wild species, although certain species have been cultivated [2]. In the genus *Annona* there are about 119 species [3]. The most famous species are *Annona cherimola* Mill (cherimoya), *Annona muricata* L. (soursop), *Annona squamosa* L. (conde fruit), *Annona reticulata* L. (custard apple), and the interspecific hybrid *Atemoya* (*A. cherimola* × *A. squamosa*). In particular, *Annona cherimola* is the most diffused specie in subtropical countries [4]. Currently, Andalusia has around 3000 hectares devoted to the production of cherimoyas, which yield around 40,000 tonnes of fruit; this makes Andalusia the world’s leading grower in terms of both production and acreage [5]. Most of Spain’s production comes from plantations in the provinces of Granada and Malaga [6]. The commercial value of cherimoya is mainly related to its fruit, essentially destined for fresh consumption. However, cherimoya fruit is traditionally used for the treatment of several diseases. Recently, it has been shown that cherimoya fruit and its by-products (leaves and seeds) are a rich source of bioactive compounds including phenolic compounds (catechin, proanthocyanidins, rutin) [7], alkaloids (annocherines, norisocorydine, cheritamine, annonaine) [5,8], acetogenins (cherimolin-2 and almunequin) [9], terpenes (myrcene, pinene, linalool, caryophillene, terpenolene and germacrene) [10,11] and cyclopeptides (cherimoya cyclopetide E and cherimoya cyclopetide F) [12]. Concretely, cherimoya leaves are currently ingredients used in traditional medicine preparations and folk teas for the treatment of gastric, intestinal, cardiovascular, skin, and eye diseases [13]. These beneficial properties are due in part to their content in phenolic compounds which possess antioxidant activities [5,14,15,16,17,18] that could be exploited by the food, nutraceutical and cosmeceutical industries. Nevertheless, the bioactive properties of these phenolic compounds in cherimoya leaves have been poorly explored. Today, these leaves are just discarded as agricultural waste, usually in considerable quantities. Therefore, the recovery of phenolic compounds from cherimoya leaves could be profitable for the food industry. In recent years, ultrasound-assisted extraction (UAE) has been an efficient extraction technique for the recovery of phenolic compounds from plants. UAE has high reproducibility, is an efficient and simplified technique, has low cost, requires short processing times and has low or no amount of solvents [19]. Ultrasound (US) facilities component extraction because it produces a phenomenon known as cavitation which causes the rupture of the cellular wall, reduces the pore size of solid materials, and provides an increase in the contact surface area between the solid phase and the solvent [20]. For this reason, the aim of the present study was the establishment, for the first time, of an optimized extraction via sonotrode ultrasound-assisted extraction to obtain the highest phenolic content from cherimoya leaves and the highest in vitro antioxidant activity by DPPH, FRAP and ABTS assays. For that purpose, a Box-Behnken design (BBD) was carried out with three independent variables (amplitude, time and % of ethanol (EtOH)). The leaf extracts were analyzed by an Ultra Performance liquid chromatography (UPLC) system coupled to an electrospray ionization (ESI) source and a quadrupole time-of-flight (qTOF) mass detector. Therefore, this study presents a new strategy to obtain cherimoya leaf extracts enriched in bioactive compounds that could be used in the food, pharmaceutical, and cosmetic industries.

## 2. Results and Discussion

### 2.1. Identification of Phenolic Compounds from Cherimoya Leaf Extracts by UPLC-ESI-TOF-MS

Cherimoya leaf extracts obtained by ultrasonic-assisted extraction by sonotrode were analyzed by UPLC-ESI-TOF-MS. Phenolic compounds were identified by using their mass spectra and by comparison with the data reported in previous studies and several databases. The analytical parameters that allowed the identification of specific phenolic compounds in cherimoya leaf extracts were the following: retention time, experimental and calculated *m*/*z*, error, Fit Conf %, main in source fragments and molecular formula [M-H]^−^. These parameters are shown in Table 1.

A total of 29 phenolic compounds were identified in cherimoya leaf extracts, eight phenolic acid derivatives and twenty-one flavonoids. Four of these compounds were identified for the first time in cherimoya leaves. Peaks 1 and 2 with retention times at 1.12 and 2.24 min with a molecular formula C_15_H_15_O_11_ showed a molecular ion at *m*/*z* 371.0614 and the fragment ions at *m*/*z* 209.0302 and 743.1304 and they were detected as caffeoyl-glucaric acid derivatives [21]. Peak 3 at 3.70 min presented a molecular ion at *m*/*z* 355.0653 and fragment ions at *m*/*z* 163.0399, 209.0293 and 711.1283 and a molecular formula C_15_H_15_O_10_ was identified as a *p*-coumaroylglucaric acid derivative [21]. Peaks 4 and 8 at 3.90 and 5.18 min which presented a molecular formula of C_15_H_13_O_6_, *m*/*z* 289.0711 and a fragment ion at *m*/*z* 245 were proposed as catechin and epicatechin, respectively [5,21]. Peaks 5, 6 and 7 with retention times at 4.55, 4.72 and 4.92 min exhibited a molecular ion at *m*/*z* 353.0873 with molecular formula C_16_H_17_O_9_ and fragment ions at *m*/*z* 191.0561 and 707.1754 were identified as chlorogenic acid isomers [21]. Peaks 9 and 11 at 5.52 and 5.72 min (C_33_H_39_O_21_) and *m*/*z* 771.1984 were tentatively identified as isomers of quercetin 3-O-rutinoside-7-O-glucoside [5,21]. Peak 10 at 5.56 min, molecular formula C_30_H_25_O_12,_ *m*/*z* 577.1324 and fragment ions at *m*/*z* 289.0706, 425.0938 was proposed to be procyanidin dimer type B [21]. Peaks 12 and 13 (5.90 and 6.07 min) with a molecular ion at *m*/*z* 337.0923 (C_16_H_17_O_8_) and fragment ions at *m*/*z* 191.05553, 163.0376, 173.0376 and 119.04448, were identified as isomers of 5-*p*-coumaroylquinic acid [22]. This compound has been identified previously in bamboo shoot shells. To our knowledge, this compound has been identified for the first time in cherimoya leaves. Peak 14 at 7.14 min at *m*/*z* 865.1959 and a molecular formula of C_45_H_37_O_18_ presented a fragment ion at *m*/*z* 577.1373, which was assigned as procyanidin trimer type B [21]; 7.65 min (peak 15) with *m*/*z* 1153.2571 (C_60_H_49_O_24_) was proposed to be procyanidin tetramer type B [21]. Peaks 16 and 17 (8.52 and 8.69 min) with a molecular formula of C_32_H_37_O_20_, *m*/*z* 741.1866 and a fragment ion at *m*/*z* 300.027, were characterized as isomers of calabricoside A [21]. Peaks 18 and 19 at 8.75 and 9.07 min presented a molecular ion at *m*/*z* 609.1456 and a molecular formula C_27_H_29_O_26_ with a fragment at *m*/*z* 300.027 and were identified as isomers of rutin [5,21,23]. At 9.20 min, the compound 20 (C_21_H_19_O_12_), *m*/*z* 463.0867 with a fragment ion at *m*/*z* 300.0269 was detected as quercetin hexoside [5,21]. Peaks 21 and 22 (C_32_H_37_O_19_) at 9.22 and 9.42 min, *m*/*z* 725.1929 and a fragment ion at *m*/*z* 284 were tentatively detected as kaempferol-lathyroside-7-O-α-l-rhamnopyranoside isomers, which have been identified previously in chickpea flour [24], thus, these compounds have been detected for the first time in cherimoya leaf extracts. The compound 23 at 9.48 min, *m*/*z* 593.1506 and a molecular formula of C_27_H_29_O_15_ with a fragment ion at *m*/*z* 285.0387 was proposed to be kaempferol 3-galactoside-7-rhamnoside [5]. Peaks 24 and 26 at 9.78 and 10.10 min with a molecular ion at *m*/*z* 447.0927 (C_21_H_19_O_11_) and a fragment ion of *m*/*z* 285 were identified as kaempferol hexoside [21]. Peak 25 at a retention time of 9.95 min, *m*/*z* 593.1508 (C_27_H_29_O_15_) presented a fragment *m*/*z* 285.0404, which corresponds with luteolin-3-galactoside-7-rhamnoside [5]. At 10.14 min, the peak 27 at *m*/*z* 505.0982 (C_23_H_21_O_13_) and fragment ions at *m*/*z* 151.0026, 301.0327, 447.094 was proposed to be quercetin hexose acetate, this compound has been detected previously in blackberry [25]. It must be emphasized that this compound has been identified for the first time in Cherimoya leaves. Finally, compounds 28 and 29 (13.50 and 13.58 min) with a molecular formula C_30_H_25_O_13_, *m*/*z* 593.1295 showed a fragment ion at *m*/*z* 447, which corresponds with isomers of kaempferol 3-O-β-D-(6^-O-p-coumaroyl) galactopyranoside [21].

### 2.2. Fitting the Model

The BBD carried out for the optimization of UAE sonotrode factors, considering the experimental values obtained for the variable responses, is exhibited in Table 2.

The regression coefficients of the models and the results of the analysis of variance (ANOVA) are shown in Table 3. The evaluation of the model was carried out according to the significance of the regression coefficients, quadratic correlation coefficients (R^2^) and lack of fit. According to previous studies, the level of significance was α < 0.1 in order to increase the number of significant variables [26]. The significant variables for the response variable of TPC were the linear effect of amplitude % (*v*/*v*) (X_1_) (*p* = 0.014629) and its quadratic effect (X_11_) (*p* = 0.016527), the quadratic effect of time (X_22_) (*p* = 0.099676), the linear effect of % EtOH (X_3_) (*p* = 0.005422) and its quadratic effect (X_33_) *p* = 0.001523) and the cross effect between amplitude and % EtOH (X_13_) (*p* = 0.092264) and time with % EtOH (X_23_) (*p* = 0.048384). The significant variables for the variable response of DPPH were the linear effect of amplitude (X_1_) (*p* = 0.018346) and its quadratic effect (X_11_) (*p* = 0.022500), quadratic effect of time (X_22_) (*p* = 0.061514) linear effect of %EtOH (X_3_) (*p* = 0.002566) and its quadratic (X_33_) (*p* = 0.001407) and the cross effect between time and % EtOH (X_23_) (*p* = 0.033680). In addition, the significant effects on the response ABTS were the following: amplitude % (*v*/*v*) (X_1_) (*p* = 0.028732) and its quadratic effect (X_11_) (*p* = 0.044629), linear effect of time (X_2_) (*p* = 0.092438), the linear effect of % EtOH (X_3_) (*p* = 0.005781) and its quadratic effect (X_33_ = 0.005475), the cross effect between time and % EtOH (X_23_) (*p* = 0.040944). Finally, the significant effects for the FRAP were the linear effect of amplitude (X_1_) (*p* = 0.016602) and its quadratic (X_11_) (*p* = 0.020975) the linear effect of time (X_2_) (*p* = 0.098882), % EtOH (X_3_) (*p* = 0.022687) and its quadratic (X_33_) (*p* = 0.010810).

An analysis of variance (ANOVA) with a 95% confidence level was generated and the effect and regression coefficients of individual linear, quadratic and interaction terms were determined. The models presented a high correlation between independent factors and response variables with quadratic correlation coefficients (R^2^) from 85.84–95.78%. According to Le Man et al. [27], a model is adequate when R^2^ > 0.75. In addition, the *p*-value of lack-of-fit was used to verify the adequacy of the model, which was non-significant (*p* > 0.05); thus, the model fits well (Table 3).

### 2.3. Analysis of Response Surfaces

**Figure 1:**
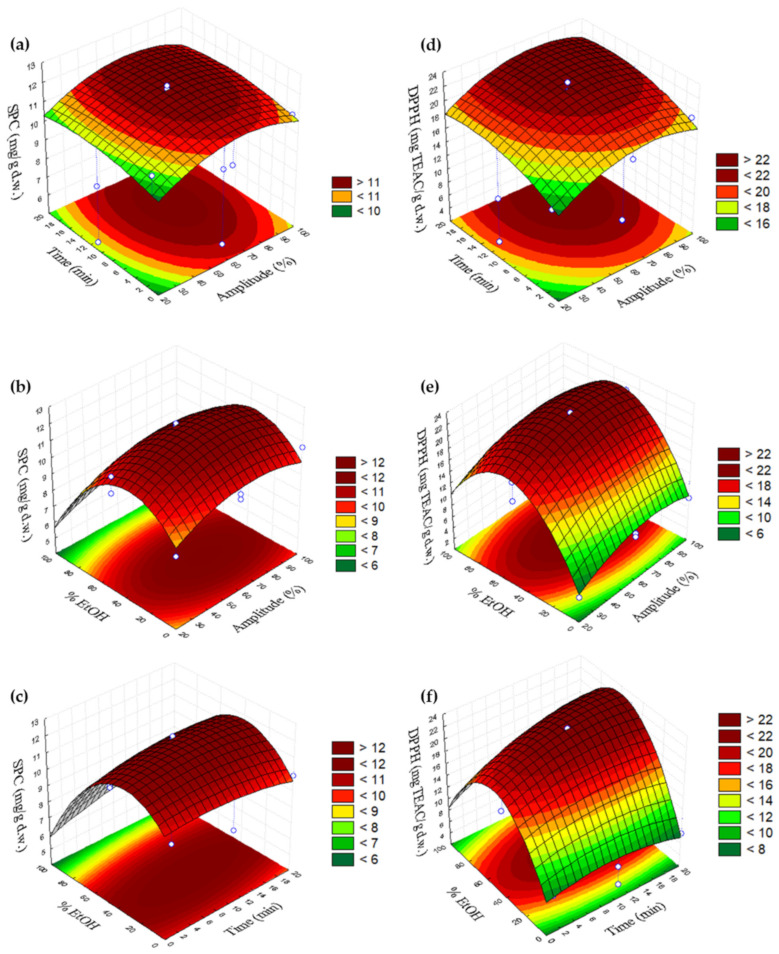
Three-dimensional plots showing the effects of % amplitude (*v*/*v*) (X_1_) with time (X_2_) (**a**,**d**), % amplitude (X_1_) with % EtOH (X_3_) (**b**,**e**) and time (X_2_) with % EtOH (X_3_) (**c**,**f**) on the sum of phenolic compounds and DPPH.

**Figure 2:**
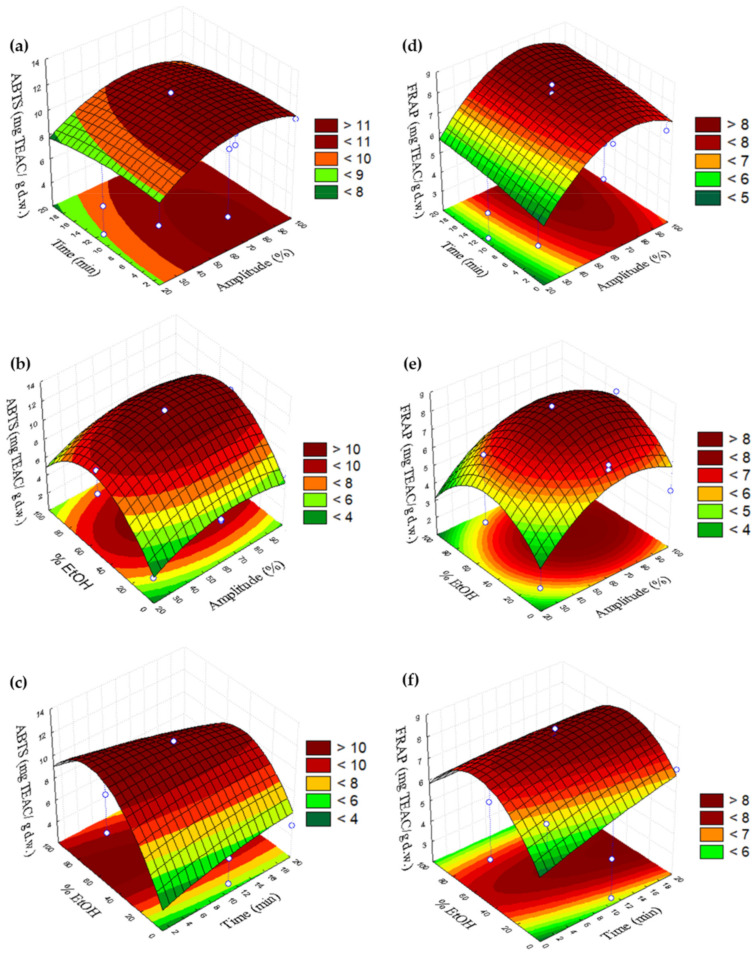
Three-dimensional plots showing the effects of % amplitude (*v*/*v*) (X_1_) with time (X_2_) (**a**,**d**), % amplitude (X_1_) with % EtOH (X_3_) (**b**,**e**) and time (X_2_) with % EtOH (X_3_) (**c**,**f**) on the ABTS and FRAP.

Figure 1 and Figure 2 are the three-dimensional plots showing the effects of % amplitude (*v*/*v*) (X_1_) with time (X_2_) (a,d), % amplitude (X_1_) with % EtOH (X_3_) (b,e) and time (X_2_) with % EtOH (X_3_) (c,f) on the sum of phenolic compounds and DPPH and ABTS and FRAP, respectively.

According to the surface response plots obtained for the content of the sum of phenolic compounds, its highest content was obtained at the following conditions: 60–80% amplitude and 8–20 min (Figure 1a), 20–50% EtOH and 50–80% amplitude (Figure 1b) and 30–60% EtOH and 4–20 min. In the case of antioxidant activities by different assays, the maximum antioxidant activity by DPPH was observed in the range of 8–20 min and 60–90% of amplitude (Figure 1d), 40–70% EtOH and 40–90% amplitude in Figure 1e and 40–60% EtOH and 8–20 min in Figure 1f. The maximum value by ABTS in Figure 2a was observed at 55–85% amplitude and 1–14 min, 40–70% EtOH and 40–100% amplitude (Figure 2b) and 40–80% EtOH and 1–20 min (Figure 2c). Finally, the highest antioxidant activity by the FRAP assay was obtained at 4–20 min and 60–90% amplitude (Figure 2d), 30–60% EtOH and 50–90% amplitude (Figure 2e) and 40–60% EtOH and 10–20 min (Figure 2f).

### 2.4. Optimization of Sonotrode Ultrasonic Assisted Extraction

#### 2.4.1. Optimal Sonotrode Ultrasonic Assisted Extraction Conditions

The optimal extraction conditions for the sonotrode were chosen based on three-dimensional response surface plots. The accuracy of the model was established by comparing the predicted with the experimental results. The optimal conditions to obtain the highest amount of sum of phenolic compounds and antioxidant activities were: 70% amplitude, 10 min and 40% EtOH to obtain the predicted values of 12.1 ± 0.6 mg/g d.w. for the sum of phenolic compounds and 21 ± 2, 11 ± 1, 8 ± 1 mg Trolox eq/g d.w. for DPPH, ABTS and FRAP, respectively.

No significant differences (*p* < 0.05) were obtained between the experimental and predictable values (Table 4). These results were compared with an ultrasonic-assisted extraction in a bath at optimum conditions previously established by a study that used 80% EtOH and 20 min (Table S1) [21]. By comparison of these results, the content in the phenolic compounds and antioxidant activities by using probe-type (sonotrode) was 2.3 and 1.6–2 times higher in comparison with the bath. Therefore, the use of a sonotrode has been shown to be more efficient than the ultrasonic-assisted extraction by a bath. This could be due to the fact that the power reached into the solvent with the sonotrode is higher than that used in the bath, improving the mass transfer of phenolic compounds across cellular membranes into the solution. In addition, the delivered intensity is attenuated by the water present in the bath and the glassware used for the experiment [28].

The optimum obtained by the sonotrode was compared with the extraction performed by ultrasonic bath according to the method described by Diaz-de-Cerio et al. [16]. The results are shown in Table 5.

As noticed, sonotrode extraction reported a higher recovery of phenolic compounds compared to ultrasonic baths. In fact, the total phenolic content in the sonotrode extract was 2.3 times higher than in the bath extract. A similar trend was reported for flavonoids (2.6 times higher) and phenolic acids (1.7 times higher).

Antioxidant activity of the extracts evaluated by DPPH, ABTS and FRAP confirmed the trend reported for the phenolic content.

#### 2.4.2. Quantification of Phenolic Compounds by HPLC-MS in Different Cultivars of Cherimoya Leaf Extracts at Optimal UAE Sonotrode Conditions

The sonotrode extraction optimum conditions established for the cultivar ‘Fino de Jete’ was applied in other cultivars of cherimoya leaves ‘Campas’, and ‘Negrito Joven’. Among 29 phenolic compounds identified in the cultivars of cherimoya leaves, 23 of them were quantified in all cultivars by HPLC-MS (except proanthocyanidins) (Table 6). Flavonoids were the most abundant phenolic compounds, which content comprise 77.3, 83.8, 76.1 and 83.1% of their total phenolic content in ‘Campas-1’, ‘Campas-2’, ‘Fino de Jete’ and ‘Negrito Joven’, respectively. These results were similar to those obtained by a previous study that reported a total amount of flavonoids between 63 and 76% regarding the total phenolic content in cherimoya leaf extracts [21]. Regarding the flavonoids, the most concentrated flavonoid in cherimoya leaf extracts was rutin. These results are in agreement with previous studies that reported rutin as the most abundant flavonoid in different cultivars of cherimoya leaves [5,21]. Concentrations of rutin represent around 40.3–43.2% of their total phenolic content for all cultivars of cherimoya leaf extracts. This result is similar to the one obtained by a previous study that reported a rutin content in *Annona muricata* leaves, which represents 37.5% of its total phenolic content [29]. In general, slight quantitative differences were found among the cultivars under study. In particular, the lowest content in rutin was obtained in the cultivar ‘Campas-2’; the value was 14.9% lower than in ‘Campas-1’and 11.8 and 9.7% lower than in the cultivars ‘Fino de Jete’ and ‘Negrito Joven’.

By comparison of the sum of phenolic compounds obtained in all cultivars, it has no shown significant differences between the cultivars ‘Campas 1’ and ‘Fino de Jete’. These results are in concordance with a previous study that reported no significant difference in the total phenolic content obtained in the cultivars ‘Fino de Jete’ and ‘Campas’ obtained in the same harvest season (650.75 and 639.4 mg gallic acid eq/g d.w.), respectively [5]. In addition, the sum of phenolic compounds obtained in ‘Negrito Joven’ and ‘Campas 2’ was slightly lower than the obtained by ‘Campas-1’ and ‘Fino de Jete’. Hence, the fact that there are almost no differences in the phenolic content could be due to these cultivars being grown under the same agronomic conditions [30].

The sum of phenolic compounds obtained in ‘Fino de Jete’ was in the same order of magnitude that the reported in the same cultivar by Diaz de Cerio et al. (2018), which reported a total content of 14 and 17 mg/g d.w. obtained in the same cultivar at 80% and 70% EtOH by using a sonicator for 20 min [21]. In addition, the phenolic content obtained in ‘Fino de Jete’ cherimoya leaves was similar to that reported in the fruit peel (20.35 mg/g d.w.) and it was double that of the pulp and the seeds (7.4 and 7.6 mg/g d.w., respectively) [7].

### 2.5. Antioxidant Activities Obtained in Cherimoya Leaves Cultivars at Optimal Conditions

Results of the antioxidant activities in the different cultivars of cherimoya leaves are shown in Table 6. According to the results, the highest antioxidant activity was obtained in ‘Fino de Jete’, ‘Negrito Joven’ and ‘Campas-1’ by DPPH, which were 5.4–9.3% higher than ‘Campas-2’. In addition, the highest antioxidant values obtained by ABTS and FRAP were obtained in ‘Fino de Jete’ and ‘Negrito Joven’, followed by ‘Campas-1’ and ‘Campas-2’. Therefore, ‘Campas-2’ possess antioxidant activity values slightly lower than the rest of the cultivars and these results agree with those obtained from the sum of compounds by HPLC-MS. The values of antioxidant activities in ‘Fino de Jete’ and ‘Campas’ (8.2–8.8, 4.4–4.8 and 2.9–3.6 mmol trolox/100 g d.w.) by DPPH, ABTS and FRAP were in the same order of magnitude than those obtained by a previous study in the same cultivars [5]. The statistical correlation between phenolic compounds and antioxidant activities is shown in Table S2. Both DPPH and ABTS exhibit a significant positive correlation with rutin and kaempferol 3-O-ß-D-(6^-O-p-coumaroyl) galactopyranoside, flavonoids and the sum of phenolic compounds. However, DPPH also possesses a significant positive correlation with calabricoside A I. In addition, ABTS and FRAP have shown significant correlations with caffeoyl-glucaric acid derivative II, p-coumaroylglucaric acid derivative, procyanidin dimer type B and lathyroside-7-O-α-Lrhamnopyranoside isomer I. Therefore, differences in correlation between specific phenolic compounds with the antioxidant activities in different assays could be due to the diverse responses of phenolic compounds to different antioxidant reaction mechanisms [31]. DPPH and ABTS neutralized the two radicals by radical quenching via hydrogen atoms or by radical quenching via hydrogen atoms. However, the FRAP essay consists of a reduction in the ferric ion by means of donor electrons in the sample [32].

### 2.6. Quantification of Proanthocyanidins in Cherimoya Leaf Extract by HPLC-FLD

The quantification of proanthocyanidins in cherimoya leaf extract was performed using HPLC-FLD (Supplementary Figure S1). The calibration curve of the standard catechin was used to quantify the proanthocyanins (y = 88.254x + 56.648, r^2^ = 0.9991). The quantification of oligomers and polymers was carried out by the application of different correction factors established by Robbins et al. [33].

The concentration values of proanthocyanidins obtained in cherimoya leaf extracts at optimum sonotrode conditions are shown in Table 7.

The total proanthocyanidins content was in the same order of magnitude as reported by Díaz-De-Cerio et al. (2018) (5.38 ± 0.03 and 5.5 ± 0.2 mg/g leaf d.w., respectively) [21]. Among the proanthocyanidins presented in the cultivars of cherimoya leaves, the most concentrated ones were the monomers (catechin and epicatechin). The highest concentration of monomers was obtained in ‘Campas-1’ and ‘Campas-2’, with values 22.0–25.9% and 72.4–77.9% higher than in ‘Fino de Jete’ and ‘Negrito Joven’. This result is in concordance with that reported by Mannino et al. (2020), which reported a concentration in monomers in ‘Campas’, which was 46.8% higher than that obtained in ‘Fino de Jete’ [5]. In addition, trimers of proanthocyanidins were found in a high concentration, which represents between 13.4 and 16.0% of their total content of proanthocianidins. There is no information regarding the content of oligomers of proanthocyanidinds in cherimoya leaves for comparative purposes. However, when compared with those described for the pulp of cherimoya, the highest content of proanthocyanidins obtained in ‘Mateus I’ was 43.5% lower than the mean obtained in the present study in cherimoya leaves [34]. The highest proanthocyanidin content was obtained in the cultivars ‘Fino de Jete’ and ‘Campas-1’, which was 15.7 and 52.2% higher than that obtained in ‘Campas-2’ and ‘Negrito Joven’. Therefore, the sonotrode could be considered an efficient extraction technique that allows a good recovery of phenolic substances from cherimoya leaf that could be easily scale-up at the industrial level.

## 3. Materials and Methods

### 3.1. Samples

‘Fino de Jete’, ‘Negrito joven’, ‘Campas-1’ and ‘Campas-2’ cherimoya leaves cultivars were harvested in the Tropical Coast of Granada (Spain). These cultivars were harvested in September 2020. Leaves were air dried at room temperature in dark conditions (25–28 °C for 1 week) and afterward, they were milled using an A10 basic miller from IKA (IKA, Staufen, Germany).

### 3.2. Chemicals

Ethanol, methanol and HPLC-grade acetonitrile were purchased from Merck KGaA (Darmstadt, Germany) and water was purified using a Milli-Q system (Millipore, Bedford, MA, USA). The acetic acid used was purchased from Fluka (Buchs, Switzerland). Vanillic acid, chlorogenic acid, quercetin, catechin and rutin were purchased from Sigma-Aldrich (St. Louis, MO, USA).

### 3.3. Experimental Design

A Box-Behnken design with three variables was carried out in order to optimize the extraction parameters to obtain the highest content of phenolic compounds in ‘Fino de Jete’ cherimoya leaves. In this study, three independent variables were evaluated: amplitude (X_1_), time (X_2_), and percentage of EtOH/H_2_O (X_3_), with three levels for each variable. The response variables (Y) were total phenolic content (TPC) obtained by HPLC-MS, and antioxidant activities by FRAP, DPPH and ABTS. The range in percentage of amplitude (20, 60 and 100%) was the same as reported in a previous study on olive leaves [35]. The range of extraction times (1, 10.5 and 20 min) was chosen based on a previous study that used 20 min with a sonicator bath in cherimoya leaves [21]. Finally, the percentage of EtOH was 0, 50 and 100%, based on previous studies that used 70, 75 and 80% EtOH, respectively [5,21]. The design consisted of 15 combinations including three center points.

Response surface methodology (RSM) is the most popular tool for modeling. In RSM, a second-order polynomial equation is always used to build the relationship between the response variables and independent variables [36]. The experimental design and the determination of optimal UAE sonotrode factors were chosen based on the highest phenolic content and antioxidant activities by using the program STATISTICA 7.0 (2002, StatSoft, Tulsa, OK, USA).

### 3.4. Extraction of Phenolic Compounds in Cherimoya Leaves

The sonotrode extraction was achieved by adding 100 mL of EtOH/H_2_O mixture to 0.25 g of cherimoya leaves [1:400 (*w*/*v*)]. The sonotrode used was UP400St (Hielscher Ultrasonics GmbH, Teltow, Germany). The ultrasound amplitude, percentage of EtOH/H_2_O and extraction time were varied according to a Box-Behnken experimental design. In addition, the ultrasonic-assisted extraction with bath (Bandelin, Sonorex, RK52, Berlin, Germany) was achieved by using the conditions previously established in the literature [21]. After that, the extracts were centrifugated at 1000× *g* for 10 min, the supernatant was collected, evaporated and reconstituted in 2 mL of methanol/water (1:1, *v*/*v*). The cherimoya leaf extracts were filtered through 0.2 μm nylon syringe filters and stored at −18 °C until their analysis.

### 3.5. Antioxidant Capacity

Three different assays were used to determine the antioxidant capacity of the extracts obtained from cherimoya leaves cultivars. In all assays, Trolox (6-hydroxy-2,5,7,8-tetramethylchromen-2-carboxylic acid) was used as the standard and the results were expressed in mg of Trolox equivalents (TE)/g of dry weight.

#### 3.5.1. DPPH Radical Scavenging

A DPPH assay was performed according to the method described by Brand-Williams. et al. [37].

#### 3.5.2. ABTS Cation Radical Scavenging

The antioxidant activity of the cherimoya leaf extracts by ABTS cation radical scavenging was conducted following the method reported by Re R. et al. [38].

#### 3.5.3. Ferric Reducing Antioxidant Power (FRAP)

The FRAP of the cherimoya leaf extract was performed by using a protocol described by Pulido et al. [39].

### 3.6. Determination of Phenolic Compounds in Cherimoya Leaf Extracts by UPLC-ESI-TOF-MS Analysis

Cherimoya leaf extracts obtained by ultrasonic-assisted extraction via the sonotrode were analyzed by the ACQUITY Ultra Performance LC system (Waters Corporation, Milford, MA, USA) coupled to an electrospray ionization (ESI) source operating in the negative mode and a time-of-flight (TOF) mass detector (Waters Corporation, Milford, MA, USA). The separation was performed by using an ACQUITY UPLC BEH Shield RP18 column (1.7 µm, 2.1 mm × 100 mm; Waters Corporation, Milford, MA, USA) at 40 °C by using the gradient conditions as Verni et al. [40] The data were processed by using MassLynx 4.1 software (Waters Corporation, Milford, MA, USA).

### 3.7. Determination of Procyanidins in Cherimoya Leaf Extracts by HPLC-FLD

The analysis of procyanidins was carried out by using an Agilent 1200 Series HPLC system (Agilent Technologies, Santa Clara, CA, USA) equipped with a binary pump delivery system, a degasser, an autosampler and a fluorescence detector (FLD). The separation was carried out by using a column Develosil Diol 100Å (250 × 4.6 mm, 5µm particle size) purchased from Phenomenex (Torrance, CA, USA). The gradient conditions were the same as those previously used by Robbins et al. [33]. A standard curve of catechin at six concentration levels from 10 to 650 µg/mL was carried out for the quantification of flavan-3-ols. In addition correction factors suggested by Robbins et al. [33] were used for quantification. The results are expressed as mg catechin equivalents (CE)/g d.w.

### 3.8. Statistical Analysis

Tukey’s honest significant differences (one-way ANOVA), *p* < 0.05, were evaluated using Statistica 8.0 software (2007, StatSoft, Tulsa, OK, USA).

## 4. Conclusions

A Box-Behnken design has been used for the first time to optimize the ultrasonic-assisted extraction via sonotrode in ‘Fino de Jete’ cherimoya leaves with the aim to obtain the highest content of phenolic compounds by HPLC-MS and the highest value of the antioxidant activities by DPPH, ABTS and FRAP. According to the results, the optimal factors were 70% amplitude, 10 min and EtOH/H_2_O (40:60, *v*/*v*). Under these conditions, the total phenolic content obtained via the sonotrode was twice more than using a conventional ultrasonic bath. These optimal conditions were applied in different cultivars of cherimoya leaves ‘Campas-1’, ‘Campas-2’, ‘Fino de Jete’ and ‘Negrito Joven’ which were grown under the same agronomic conditions. In addition, the content of proanthocyanidins in these cherimoya leaf extracts was analyzed via HPLC-FLD. Cherimoya leaf extracts possess a high content in monomers, dimers, trimers and polymers. No significant differences in the phenolic and proanthocyanidins content have been obtained between ‘Fino de Jete’, and ‘Campas-1’; the content was slightly higher in comparison with ‘Negrito Joven’ and ‘Campas-2’. Therefore, the employment of this sonotrode extraction technique under the optimum conditions established could be implemented at an industrial scale to obtain a cherimoya leaf extract rich in phenolic compounds. This consists of additional investment in equipment and skilled labor but it could improve the recovery of these valuable compounds.

## Figures and Tables

**Table 1:** Identification table of phenolic compounds in cherimoya leaves obtained at UAE sonotrode conditions.

	RT (min)	*m*/*z* Experimental	*m*/*z* Calculated	Tolerance (ppm)	Error (ppm)	Fit Conf %	Fragments	Molecular Formula	Compound
1	1.12	371.0609	371.0614	20	−1.3	99.72	209.0302, 743.1304	C_15_H_15_O_11_	Caffeoyl-glucaric acid derivative I
2	2.24	371.0625	371.0614	20	3	99.96	209.0305, 743.1282	C_6_H_7_O_7_	Caffeoyl-glucaric acid derivative II
3	3.70	355.0653	355.0665	20	−3.4	94.57	163.0399, 209.0293, 711.1283	C_15_H_15_O_10_	p-coumaroylglucaric acid derivative
4	3.90	289.0711	289.0712	20	−0.7	99.98	245.0825	C_15_H_13_O_6_	Catechin
5	4.55	353.087	353.0873	20	−0.8	96.98	191.0561, 707.1754	C_16_H_17_O_9_	Chlorogenic acid I
6	4.72	353.0876	353.0873	20	0.8	95.86	191.0564, 707.1786	C_16_H_17_O_9_	Chlorogenic acid II
7	4.92	353.0876	353.0873	20	0.8	93.35	191.0564, 707.1786	C_16_H_17_O_9_	Chlorogenic acid III
8	5.18	289.0711	289.0712	20	−0.3	100	245.0806	C_15_H_13_O_6_	Epicatechin
9	5.52	771.1967	771.1984	20	−3.2	90.37		C_33_H_39_O_21_	Quercetin 3-O-rutinoside-7-O-glucoside I
10	5.56	577.1324	577.1346	20	−3.8	99.71	289.0706, 425.0938	C_30_H_25_O_12_	Procyanidin dimer type B
11	5.72	771.1959	771.1984	20	4.5	91.28		C_33_H_39_O_21_	Quercetin 3-O-rutinoside-7-O-glucoside II
12	5.90	337.0927	337.0923	20	1.2	99.85	191.05553, 163.0376, 173.0376, 119.04448	C_16_H_17_O_8_	5-p-coumaroylquinic acid I
13	6.07	337.093	337.0923	20	2.1	99.77	191.0559, 163.0386, 173.0435, 119.0499	C_16_H_17_O_8_	5-p-coumaroylquinic acid II
14	7.14	865.1959	865.198	20	−2.4	99.72	577.1373	C_45_H_37_O_18_	Procyanidin trimer type B I
15	7.65	1153.2571	1153.2614	20	−3.7	86.2		C_60_H_49_O_24_	Procyanidin tetramer type B I
16	8.52	741.1866	741.1878	20	−1.6	89.54	300.027	C_32_H_37_O_20_	Calabricoside A I
17	8.69	741.1855	741.1878	20	−3.1	99.97	300.027	C_32_H_37_O_20_	Calabricoside A II
18	8.75	609.1457	609.1456	20	0.2	99.36	300.0271	C_27_H_29_O_26_	Rutin I
19	9.07	609.1459	609.1456	20	0.5	97.64	300.0274	C_27_H_29_O_26_	Rutin II
#	RT (min)	*m*/*z* experimental	*m*/*z* calculated	Tolerance (ppm)	Error (ppm)	Fit Conf %	Fragments	Molecular formula	Compound
20	9.20	463.0867	463.0877	20	−2.2	94.75	300.0269	C_21_H_19_O_12_	Quercetin hexoside
21	9.22	725.1923	725.1929	20	−0.8	99.36	284.0324	C_32_H_37_O_19_	Kaempferol-lathyroside-7-O-α-L-rhamnopyranoside isomer I
22	9.42	725.1901	725.1929	20	−3.9	99.98	284.0315	C_32_H_37_O_19_	Kaempferol-lathyroside-7-O-α-L-rhamnopyranoside isomer II
23	9.48	593.1503	593.1506	20	−0.5	93.29	285.0387	C_27_H_29_O_15_	Kaempferol 3-galactoside-7-rhamnoside
24	9.78	447.0934	447.0927	20	1.6	88.65	285.0397	C_21_H_19_O_11_	Kaempferol hexoside I
25	9.95	593.1508	593.1506	20	0.3	93.69	285.0404	C_27_H_29_O_15_	Luteolin-3-galactoside-7-rhamnoside
26	10.10	447.0919	447.0927	20	−1.8	99.99	285.0372	C_21_H_19_O_11_	Kaempferol hexoside II
27	10.14	505.0966	505.0982	20	−3.2	99.99	151.0026, 301.0327, 447.0947	C_23_H_21_O_13_	Quercetin hexose actetate
28	13.50	593.1283	593.1295	20	−2	94.19	447.0889	C_30_H_25_O_13_	Kaempferol 3-O-β-D-(6^-O-p-coumaroyl) galactopyranoside I
29	13.58	593.1263	593.1295	20	−5.4	48.12	447.0932	C_30_H_25_O_13_	Kaempferol 3-O-β-D-(6^-O-p-coumaroyl) galactopyranoside II

**Table 2:** Experimental Box-Behnken design (BBD), with natural and coded values for the factors, and response variable values.

Run	Dependent Factors			Response Variables			
	X_1_	X_2_	X_3_	SPC	DPPH	ABTS	FRAP
1	20	1.0	50	10.96	14.77	6.86	3.81
2	100	1.0	50	10.76	18.75	10.24	6.53
3	20	20.0	50	9.94	18.03	9.40	7.52
4	100	20.0	50	10.35	20.86	9.88	7.87
5	20	10.5	0	8.47	4.48	2.84	2.33
6	100	10.5	0	10.64	7.55	5.16	4.23
7	20	10.5	100	5.47	10.82	5.15	3.62
8	100	10.5	100	6.18	13.34	6.11	7.20
9	60	1.0	0	9.60	8.37	4.83	7.29
10	60	20.0	0	9.92	7.75	4.94	7.03
11	60	1.0	100	5.60	8.44	10.27	5.55
12	60	20.0	100	8.00	15.93	3.99	3.31
13	60	10.5	50	11.55	21.71	10.51	7.67
14	60	10.5	50	11.87	23.11	11.81	8.14
15	60	10.5	50	12.02	21.88	10.92	8.55

Amplitude (%), X_2_: time (min) and X_3_: %EtOH. SPC (sum of phenolic compounds by HPLC-MS) was expressed as mg g^−1^ d.w. DPPH, ABTS and FRAP were expressed as mg trolox eq. g^−1^ d.w.

**Table 3:** Regression coefficients and analysis of variance (ANOVA) of the model for the response variables.

	SPC	DPPH	ABTS	FRAP
β_0_	7.253700 *	−1.08121	−2.89394	−0.467654
Linear				
β_1_	0.085998 *	0.24544 *	0.17005 *	0.150340 *
β_2_	0.022230	0.34860	0.32289 **	0.206726 **
β_3_	0.092866 **	0.43362 *	0.25189 *	0.083568 *
Cross product				
β_12_	0.000402	−0.00076	−0.00191	−0.001553
β_13_	−0.000182 **	−0.00007	−0.00017	0.000211
β_23_	0.001098 *	0.00427 *	−0.00336 *	−0.001044
Quadratic				
β_11_	−0.000595 *	−0.00163 *	−0.00099 *	−0.000982 *
β_22_	−0.004019 *	−0.01692 **	−0.00440	−0.001318
β_33_	−0.001269 *	−0.00423 *	−0.00187 *	−0.000882 *
R^2^	0.94493	0.95784	0.92139	0.85839
p (Lack of fit)	0.074024	0.146656	0.192242	0.058427

* Significant at *p* < 0.05 level, ** Significant at *p* < 0.1 level.

**Table 4:** Optimal conditions for UAE sonotrode extraction.

Optimal Conditions	SPC	DPPH	ABTS	FRAP
Amplitude %	70	70	70	70
Time (min)	10	10	10	10
%EtOH	40	40	40	40
Predicted	12.1 ± 0.6	21 ± 2	11 ± 1	8 ± 1
Observed	13.4 ± 0.7	21.9 ± 0.4	12.2 ± 0.3	8.99 ± 0.07
Significant differences	N.S.	N.S.	N.S.	N.S.
*p* value	0.1023	0.0998	0.1273	0.1109

N.S.: no significant differences. SPC was expressed as mg/g d.w. DPPH, ABTS and FRAP were expressed as mg trolox/g sample d.w.

**Table 5:** Concentration of phenolic compounds (mg/g d.w.) obtained at optimum sonotrode conditions and with ultrasonic bath in the ‘Fino de Jete’ cherimoya leaves by HPLC-MS.

Phenolic Compounds	Fino de Jete	
	Sonotrode	Bath
Caffeoyl-glucaric acid derivative I ^1^	0.225 ± 0.004 ^a^	0.13 ± 0.01 ^b^
Caffeoyl-glucaric acid derivative II ^1^	0.141 ± 0.002 ^a^	0.064 ± 0.003 ^b^
p-coumaroylglucaric acid derivative ^1^	0.36 ± 0.01 ^a^	0.198 ± 0.005 ^b^
Chlorogenic acid I ^1^	0.420 ± 0.007 ^a^	0.198 ± 0.001 ^b^
Chlorogenic acid II ^1^	0.59 ± 0.06 ^a^	0.162 ± 0.002 ^b^
Chlorogenic acid III ^1^	0.2100 ± 0.0005 ^b^	0.64 ± 0.02 ^a^
Quercetin 3-O-rutinoside-7-O-glucoside I ^2^	0.007 ± 0.001 ^b^	0.203 ± 0.005 ^a^
Quercetin 3-O-rutinoside-7-O-glucoside II ^2^	0.033 ± 0.004	<LOQ
5-p-coumaroylquinic acid I ^1^	0.69 ± 0.01 ^a^	0.49 ± 0.3 ^b^
5-p-coumaroylquinic acid II ^1^	0.54 ± 0.03 ^a^	0.0041 ± 0.0005 ^b^
Calabricoside A I ^2^	0.65 ± 0.05 ^a^	0.103 ± 0.001 ^b^
Calabricoside A II ^2^	0.66 ± 0.04 ^a^	0.027 ± 0.001 ^b^
Rutin I ^2^	2.3 ± 0.1 ^a^	1.32 ± 0.05 ^b^
Rutin II ^2^	3.1 ± 0.1 ^a^	1.8 ± 0.3 ^b^
Quercetin hexoside ^2^	1.60 ± 0.09 ^a^	0.029 ± 0.2 ^b^
Kaempferol-lathyroside-7-O-α-L-rhamnopyranoside isomer I ^3^	0.078 ± 0.009 ^a^	0.020 ± 0.001 ^b^
Kaempferol-lathyroside-7-O-α-L-rhamnopyranoside isomer II ^3^	0.038 ± 0.01 ^a^	0.015 ± 0.003 ^b^
Kaempferol 3-galactoside-7-rhamnoside ^3^	0.26 ± 0.02 ^a^	0.116 ± 0.005 ^b^
Kaempferol hexoside I ^3^	0.117 ± 0.007 ^a^	0.009 ± 0.001 ^b^
Luteolin-3-galactoside-7-rhamnoside ^3^	0.41 ± 0.01 ^a^	0.13 ± 0.02 ^b^
Kaempferol hexoside II ^3^	0.145 ± 0.008 ^a^	0.0064 ± 0.0002 ^b^
Quercetin hexose acetate ^2^	0.268 ± 0.001 ^a^	0.034 ± 0.001 ^b^
Kaempferol 3-O-ß-D-(6^-O-p-coumaroyl) galactopyranoside I ^3^	0.54 ± 0.03 ^a^	0.013 ± 0.001 ^b^
Kaempferol 3-O-ß-D-(6^-O-p-coumaroyl) galactopyranoside II ^3^	<LOQ	<LOQ
Sum flavonoids	10.2 ± 0.5 ^a^	3.9 ± 0.2 ^b^
Sum phenolic acid derivatives	3.2 ± 0.1 ^a^	1.89 ± 0.06 ^b^
Sum	13.4 ± 0.7 ^a^	5.8 ± 0.3 ^b^
DPPH	21.9 ± 0.4 ^a^	13.8 ± 0.3 ^b^
ABTS	12.2 ± 0.3 ^a^	6.72 ± 0.02 ^b^
FRAP	8.99 ± 0.07 ^a^	4.4 ± 0.3 ^b^

^1^ chlorogenic acid equivalent, ^2^ rutin equivalent, ^3^ kaempferol 3-rhamnoside equivalent. Different letters in the same line indicate significant differences among the extractions. <LOQ: Less than the limit of quantification.

**Table 6:** Quantification table of phenolic compounds and antioxidant activities in different cultivars of cherimoya leaves obtained at optimum UAE sonotrode conditions.

Phenolic Compounds	‘Campas-1’	‘Campas-2’	‘Fino de Jete’	‘Negrito Joven’
Caffeoyl-glucaric acid derivative I ^1^	0.211 ± 0.006 ^b,c^	0.201 ± 0.001 ^c^	0.225 ± 0.004 ^b^	0.336 ± 0.002 ^a^
Caffeoyl-glucaric acid derivative II ^1^	0.1337 ± 0.0005 ^b^	0.140 ± 0.001 ^b^	0.141 ± 0.002 ^b^	0.200 ± 0.003 ^a^
p-coumaroylglucaric acid derivative ^1^	0.334 ± 0.003 ^a^	0.25 ± 0.02 ^b^	0.36 ± 0.01 ^a^	0.1954 ± 0.0006 ^c^
Chlorogenic acid I ^1^	0.599 ± 0.002 ^a^	0.18 ± 0.02 ^c^	0.420 ± 0.007 ^b^	0.148 ± 0.002 ^c^
Chlorogenic acid II ^1^	0.58 ± 0.02 ^a^	0.51 ± 0.06 ^a^	0.59 ± 0.06 ^a^	0.53 ± 0.01 ^a^
Chlorogenic acid III ^1^	0.191 ± 0.005 ^b^	0.155 ± 0.007 ^c^	0.2100 ± 0.0005 ^a^	0.154 ± 0.002 ^c^
Quercetin 3-O-rutinoside-7-O-glucoside I ^2^	0.008 ± 0.001 ^b^	0.006 ± 0.002 ^b^	0.007 ± 0.001 ^b^	0.035 ± 0.001 ^a^
Quercetin 3-O-rutinoside-7-O-glucoside II ^2^	0.078 ± 0.001 ^b^	0.077 ± 0.006 ^b^	0.033 ± 0.004 ^c^	0.208 ± 0.002 ^a^
5-p-coumaroylquinic acid I ^1^	0.57 ± 0.03 ^b^	0.22 ± 0.02 ^c^	0.69 ± 0.01 ^a^	0.293 ± 0.007 ^c^
5-p-coumaroylquinic acid II ^1^	0.41 ± 0.01 ^b^	0.133 ± 0.003 ^d^	0.54 ± 0.03 ^a^	0.204 ± 0.004 ^c^
Calabricoside A I ^2^	0.624 ± 0.008 ^a^	0.42 ± 0.01 ^b^	0.65 ± 0.05 ^a^	0.33 ± 0.02 ^b^
Calabricoside A II ^2^	0.85 ± 0.01 ^a^	0.47 ± 0.01 ^c^	0.66 ± 0.04 ^b^	0.473 ± 0.008 ^c^
Rutin I ^2^	2.18 ± 0.02 ^a^	1.90 ± 0.06 ^b^	2.3 ± 0.1 ^a^	2.1664 ± 0.0007 ^a,b^
Rutin II ^2^	3.42 ± 0.07 ^a^	2.864 ± 0.001 ^b^	3.1 ± 0.1 ^a,b^	3.11 ± 0.04 ^a,b^
Quercetin hexoside ^2^	1.19 ± 0.03 ^b^	1.30 ± 0.09 ^b^	1.60 ± 0.09 ^a^	1.763 ± 0.004 ^a^
Kaempferol-lathyroside-7-O-α-L-rhamnopyranoside isomer I ^3^	0.081 ± 0.003 ^a^	0.03 ± 0.01 ^b^	0.078 ± 0.009 ^a^	<LOQ
Kaempferol-lathyroside-7-O-α-L-rhamnopyranoside isomer II ^3^	0.06 ± 0.01 ^a^	0.015 ± 0.003 ^b^	0.038 ± 0.01 ^a,b^	0.0099 ± 0.0001 ^b^
Kaempferol 3-galactoside-7-rhamnoside ^3^	0.28 ± 0.04 ^a^	0.31 ± 0.02 ^a^	0.26 ± 0.02 ^a^	0.33 ± 0.01 ^a^
Kaempferol hexoside I ^3^	0.079 ± 0.006 ^b^	0.15 ± 0.03 ^a^	0.117 ± 0.007 ^a,b^	0.118 ± 0.004 ^a,b^
Luteolin-3-galactoside-7-rhamnoside ^3^	0.50 ± 0.04 ^b^	0.47 ± 0.06 ^b^	0.41 ± 0.01 ^b^	0.68 ± 0.03 ^a^
Kaempferol hexoside II ^3^	0.097 ± 0.006 ^c^	0.164 ± 0.005 ^b^	0.145 ± 0.008 ^b^	0.209 ± 0.003 ^a^
Quercetin hexose acetate ^2^	0.25 ± 0.04 ^a^	0.117 ± 0.008 ^b^	0.268 ± 0.001 ^a^	0.238 ± 0.008 ^a^
Kaempferol 3-O-ß-D-(6^-O-p-coumaroyl) galactopyranoside I ^3^	0.458 ± 0.006 ^b^	0.94 ± 0.07 ^a^	0.54 ± 0.03 ^b^	0.48 ± 0.09 ^b^
Kaempferol 3-O-ß-D-(6^-O-p-coumaroyl) galactopyranoside II ^3^	<LOQ	<LOQ	<LOQ	<LOQ
Sum flavonoids	10.2 ± 0.3 ^a^	9.24 ± 0.08 ^b^	10.2 ± 0.5 ^a^	10.15 ± 0.03 ^a^
Sum phenolic acid derivatives	3.03 ± 0.02 ^a^	1.8 ± 0.1 ^c^	3.2 ± 0.1 ^a^	2.06 ± 0.03 ^b^
Sum	13.2 ± 0.3 ^a^	11.03 ± 0.04 ^c^	13.4 ± 0.7 ^a^	12.208 ± 0.002 ^b^
DPPH	21.9 ± 0.2 ^a^	20.5 ± 0.1 ^b^	21.9 ± 0.4 ^a^	22.4 ± 0.2 ^a^
ABTS	11.61 ± 0.01 ^b^	11.11 ± 0.08 ^c^	12.2 ± 0.3 ^a^	12.53 ± 0.01 ^a^
FRAP	7.4 ± 0.5 ^b^	7.24 ± 0.03 ^b^	8.99 ± 0.07 ^a^	9.8 ± 0.1 ^a^

^1^ chlorogenic acid equivalent, ^2^ rutin equivalent, ^3^ kaempferol 3-rhamnoside equivalent, The results are expressed in mg/g d.w. DPPH, ABTS and FRAP values are expressed as mg Trolox eq/g d.w. Different letters in the same line indicate significant differences among the cultivars. <LOQ: Less than limit of quantification.

**Table 7:** Table of quantification of proanthocyanidins (mg/g d.w.) in different cultivars of cherimoya leaves at optimum sonotrode conditions by HPLC-FLD.

Proanthocyanidins	‘Campas-1’	‘Campas-2’	‘Fino de Jete’	‘Negrito Joven’
monomers	5.00 ± 0.01 ^a^	5.16 ± 0.02 ^a^	4.098 ± 0.003 ^b^	2.9 ± 0.2 ^c^
dimers	1.7 ± 0.2 ^a^	2.2 ± 0.2 ^a^	1.81 ± 0.02 ^a^	0.87 ± 0.01 ^b^
dp3	3.2 ± 0.1 ^a^	2.3 ± 0.3 ^b^	2.78 ± 0.02 ^a^	1.56 ± 0.05 ^c^
dp4	2.8 ± 0.3 ^a^	1.37 ± 0.01 ^d^	2.31 ± 0.05 ^b^	1.54 ± 0.02 ^c^
dp5	1.94 ± 0.04 ^b^	2.5 ± 0.2 ^a^	1.44 ± 0.02 ^c^	0.89 ± 0.01 ^d^
dp6	1.13 ± 0.02 ^a^	0.45 ± 0.01 ^c^	0.67 ± 0.05 ^b^	0.33 ± 0.04 ^d^
dp7	0.72 ± 0.01 ^b^	0.27 ± 0.06 ^d^	0.67 ± 0.01 ^c^	0.88 ± 0.01 ^a^
dp8	0.50 ± 0.05 ^a^	0.081 ± 0.002 ^c^	0.32 ± 0.05 ^b^	0.33 ± 0.07 ^b^
dp9	0.218 ± 0.001 ^a^	0.027 ± 0.002 ^c^	0.104 ± 0.006 ^b^	0.112 ± 0.005 ^b^
dp10	0.168 ± 0.003 ^a^	0.017 ± 0.001 ^d^	0.086 ± 0.007 ^c^	0.106 ± 0.001 ^b^
dp11	0.08 ± 0.01 ^a^	<LOQ	0.028 ± 0.005 ^c^	0.060 ± 0.001 ^b^
dp12	0.047 ± 0.002 ^a^	<LOQ	0.0052 ± 0.0002 ^c^	0.017 ± 0.001 ^b^
dp13	0.0094 ± 0.0001 ^a^	N.D.	N.D.	0.00347 ± 0.00001 ^b^
dp14	0.00439 ± 0.00005	N.D.	N.D.	N.D.
polymer	2.3 ± 0.2 ^b^	2.9 ± 0.5 ^b^	5.14 ± 0.05 ^a^	1.73 ± 0.02 ^c^
Total	19.9 ± 0.4 ^a^	17.2 ± 0.4 ^b^	19.45 ± 0.02 ^a^	11.3 ± 0.2 ^c^

dp = degree of polymerization. <LOQ: Less than limit of quantification. N.D.: Not detected. Different letters in the same line indicate significant differences among the cultivars. <LOQ: Less than limit of quantification

## Data Availability

Not applicable.

## Supplementary Materials

The following supporting information can be downloaded at: https://www.mdpi.com/article/10.3390/plants11152034/s1, Table S1. Correlation analysis of phenolic content and antioxidant activities of Cherimoya leaf extracts. Figure S1. Chromatographic separation of proanthocyanidins by HPLC-FLD.
