# Supplementary material for: Development of an Effective Sonotrode Based Extraction Technique for the Recovery of Phenolic Compounds with Antioxidant Activities in Cherimoya Leaves
Source: Plants (Basel). 2022 Aug 4;11(15):2034. doi: 10.3390/plants11152034 (PMC9370491; doi:10.3390/plants11152034)
Supplement: Supplementary file 1 [file plants-11-02034-s001.zip › plants-1837686-supplementary.pdf]

Supplementary Table S1. Correlation analysis of phenolic content and antioxidant activities of Cherimoya leaf extracts.

	DPPH	ABTS	FRAP
Caffeoyl-glucaric acid derivative I	-0.58	-0.23	0.02
Caffeoyl-glucaric acid derivative II	0.65	0.81*	0.85*
p-Coumaroylglucaric acid derivative	0.52	0.72*	0.79*
Catechin	0.04	-0.13	-0.29
Chlorogenic acid I	0.37	0.53	0.60
Chlorogenic acid II	0.25	-0.11	-0.35
Chlorogenic acid III	0.22	0.23	0.01
Epicatechin	0.30	0.15	0.01
Quercetin 3-O-rutinoside-7-O-glucoside I	0.25	0.44	0.55
Procyanidin dimer type B	0.57	0.73*	0.75*
Quercetin 3-O-rutinoside-7-O-glucoside II	-0.23	-0.05	0.08
5-p-Coumaroylquinic acid I	0.43	0.53	0.56
5-p-Coumaroylquinic acid II	0.43	0.26	0.09
Procyanidin trimer type B	0.44	0.31	0.13
Procyanidin tetramer type B	-0.68	-0.43	-0.25
Calabricoside A I	-0.75*	-0.53	-0.35
Calabricoside A II	0.07	-0.11	-0.30
Rutin I	0.28	-0.06	-0.34
Rutin II	0.75*	0.75*	0.54
Quercetin hexoside	0.53	0.28	-0.05
lathyroside-7-O-α-Lrhamnopyranoside isomer I	0.51	0.86*	0.93*
lathyroside-7-O-α-Lrhamnopyranoside isomer II	-0.03	-0.25	-0.44
Kaempferol 3-galactoside-7-rhamnoside	0.17	-0.13	-0.43
Kaempferol hexoside I	-0.11	0.14	0.14
Luteolin-3-Galactoside-7-Rhamnoside	-0.58	-0.20	0.00
Kaempferol hexoside II	0.43	0.51	0.47
Quercetin hexose actetate	0.08	0.48	0.65
Kaempferol 3-O-ß-D-(6^-O-p-coumaroyl) galactopyranoside I	0.84*	0.72*	0.52
Kaempferol 3-O-ß-D-(6^-O-p-coumaroyl) galactopyranoside II	-0.91*	-0.71*	-0.53
Flavonoids	0.73*	0.89*	0.70
Phenolic acid derivatives	0.44	0.18	-0.06
Sum of phenolic compounds	0.79*	0.82*	0.57

Results are expressed as Pearson correlation coefficients with indicated level of significance. $^{*}p$<0.05; DPPH= 2,2-diphenyl-1-picrylhydrazyl, FRAP= The ferric reducing antioxidant power; ABTS: 2,2′-azino-di (3-ethylbenzothiazoline)-6-sulfonic acid.

Supplementary Figure S1. Separation of proanthocyanidins by HPLC-FLD.

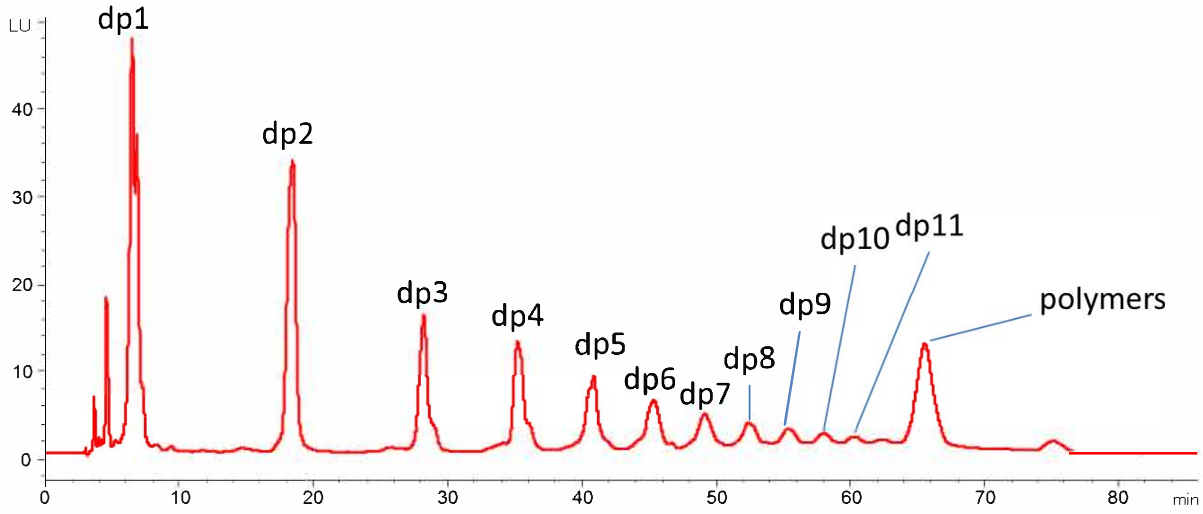

dp = degree of polymerization
